# The Patient's Experience of Working with Multiple Allied Health Professional Students – A Qualitative Interview Study

**DOI:** 10.1177/23743735241241461

**Published:** 2024-04-28

**Authors:** Adele Anderson, Julie Morrow, Anita Knighton, Andrew Lloyd, Jane Noble, Gemma Bradley

**Affiliations:** 1 Adele Anderson, Advanced Specialist Physiotherapist, 2268 North Cumbria Integrated Care NHS Foundation Trust, Newcastle-Upon-Tyne, UK; 2 Julie Morrow, AHP Consultant and Deputy Director AHP and Psychological Services, Cumbria, 5633 Northumberland, Tyne and Wear Foundation NHS Trust, Newcastle-Upon-Tyne, UK; 3 Anita Knighton, Contributor, Cumbria, Northumberland, Tyne and Wear Foundation NHS Trust Involvement Bank, Newcastle-Upon-Tyne, UK; 4 Andrew Lloyd, Physiotherapy Practice Education Facilitator, Department of Sport, Exercise and Rehabilitation, 5995 Northumbria University at Newcastle, Newcastle-Upon-Tyne, UK; 5 Jane Noble, Contributor, Cumbria, Northumberland, Tyne and Wear Foundation NHS Trust Involvement Bank, Newcastle-Upon-Tyne, UK; 6 Gemma Bradley, Assistant Professor, Department of Social Work, Education and Community Wellbeing, 5995 Northumbria University at Newcastle, Newcastle-Upon-Tyne, UK

**Keywords:** Practice education, coaching, peer-learning, collaborative learning, multiple students, patient experience

## Abstract

There are increasing numbers of learners in clinical settings as part of approaches to meet workforce demands. As a result, patients are now working with multiple learners at the same time, yet little is known about how people experience this. The aim of this study was to explore the patient experience of working with multiple allied health professional students. Structured interviews were carried out with 22 patients across hospital wards in one hospital in the North-West of England. Data was analysed using thematic analysis and four themes were identified: *consent to work with multiple students*; *responses to working with multiple students; multiple students and feelings of safety; making connections with multiple students.* Findings indicated that patients experienced positive relationships and feelings of safety with groups of students. However, patients were given limited advance or tailored information about working with a group of students which is an important area to address.

## Key Points

1. There are increasing numbers of learners in healthcare settings yet limited research which aims to understand how patients experience working with multiple students at the same time.
2. The findings of this qualitative study suggest patients experience positive relationships and feelings of safety.
3. However, patients were not always given advance notice of working with a group of students and they did not receive tailored information about why working with a group of students was part of their care.

## Introduction

There are increasing numbers of learners in health and care settings. As a result, patients are working with multiple learners at the same time, yet little is known about how people experience this. The aim of this study was to explore the patient experience of working with multiple allied health professional students. We use the word ‘patient’ when discussing the design of this study and in the presentation of findings as all participants were on inpatient wards at the time of their involvement.

Health and Care workforce shortages are recognised at an international level (1). In the United Kingdom (UK), the number of staff trained has not kept pace with workforce demands with recent figures highlighting 112 000 vacancies in the National Health Service (2). The NHS Long Term Workforce Plan (2) sets clear priorities for increasing and diversifying the health and care workforce to meet future demands. Strategies to address long-term workforce needs include increasing University training places for healthcare students across professions including medicine, nursing and allied health professions (AHPs), alongside new qualifications such as apprenticeships, and development of new roles such as physician associates. For AHPs, there is a commitment to grow training places to 18 800 by 2031/32, representing a 25% increase from current numbers (2).

Increasing numbers of learners and resulting demand for placements is happening within an already pressurised placement system, influenced by widely reported workforce shortages and vacancies (3), and following a period of placement disruption and catch-up requirements during different phases of the COVID-19 pandemic. Higher Education Institutions (HEIs) and healthcare teams are being encouraged to develop new models of practice-learning to cope with increased student numbers. One such change is a move away from one-to-one models, which have been dominant in nursing and AHPs (4,5), to supporting multiple learners.

Underpinning many of these models are principles of ‘Coaching’ such as empowering students to take responsibility for learning and allowing learners to work in a self-directed way to solve practice-based problems. The principles of a coaching approach act as enablers to multiple students working together in peer-learning (5) and for educators to move away from direct supervision of one student which underpins more traditional placement models.

There are examples, across multiple disciplines, of studies which evaluate the staff and student experiences of placements supporting multiple students (6,7). Although examples report different dimensions of experience including enablers, challenges, and perceived benefits, there are promising themes which include perceived increased autonomy and self-awareness among students, and increased feelings of satisfaction among staff (8). However, student experiences have also reported competition for learning opportunities and a sense of over-crowding (9). It is important to understand whether such concerns are also reflected in the patient experience.

In contrast to student and staff experiences, the patient experience of working with multiple students is not commonly reported. Anecdotal feedback from different sources, including educators, service users and carers suggests perceived concerns about how multiple students, with less directive models of supervision, potentially impacts on patient experience. Yet examples of studies reporting the patient experience of working with multiple students suggest people perceive being given more time, being satisfied with their experience and feel teams of students can improve their care (10).

To summarise, growing numbers of learners in healthcare settings is a contemporary and significant issue for services around the world, and there is little current understanding about how patients are experiencing this when they are receiving care or accessing services. The importance of patient voice and participation in undergraduate health professional education has been emphasised, with recognition that this element can often be lacking (11). Understanding how patients experience working with multiple students can make an important contribution to student and educator approaches and designing placements to support multiple students.

## Method

Qualitative research is appropriate where the issue is emerging, complex or ambiguous (12). A qualitative research design was employed to explore individual experiences of people who had worked with more than one AHP student at the same time. The study has been reported following COREQ guidelines for reporting qualitative research (13) (see Supplementary File 1), an accepted structure to assist authors in the reporting of studies, and readers to understand decisions made throughout the research process.

A research steering group met regularly throughout the research project. The steering group consisted of a service user, a carer, two occupational therapists and two physiotherapists (one each from academia and clinical practice). Five of the members were female and one was male. Initially, this group collaboratively developed the research question: how do patients experience working with a group of allied health professional students at the same time during a hospital admission? The group also collaboratively developed inclusive and accessible participant information.

The structured interview schedule was developed through an iterative process involving all group members. A Speech and Language Therapist co-designed the final version to include accessible language and visual cues for questions. The schedule was piloted with the first group of participants, with interviews carried out by the same interviewers as planned for all other interviews in the study. Feedback from the pilot interviews was subsequently discussed by the research steering group. Because no changes to the wording or structure of questions were made on review, and the interviewers remained the same, data from the first group of pilot participants was included in the final analysis.

The interview schedule included both closed and open questions – closed questions to identify a specific aspect of experience (for example, did anyone tell you more than one student might care for you at the same time?) and open questions to develop detail and examples (for example, how did you feel when you met the students?).

Although issues of consent are not unique to working with multiple students, questions about this aspect were perceived as particularly important to the service user and carer members of the research steering group. The interview therefore started with questions about consent to work with students more generally, before more specific questions about consent to work alongside a group of students. Other topics covered within the interview included communication, involvement and feelings of safety. Because little was known about this area, rather than themes or topics being developed from existing theory or evidence, topics were instead generated from the range of experiences within the group. A copy of the full interview schedule can be viewed as Supplementary File 2.

### Study Setting and Recruitment

Participants were recruited from one NHS Trust in the North-West of England, and more specifically from services who were hosting multiple AHP students on placements at the same time. Purposive sampling was used to approach people on Elderly Care wards, Acute Medicine wards and Neuro-rehabilitation wards who experienced involvement from a pair or group of AHP students during their inpatient stay. Patients were excluded from the study if the clinical team identified they did not have capacity to consent to involvement in the research.

Patients were approached by a member of the research team (AA) who verbally explained the research project and provided a written participant information sheet. The information sheet included information such as the reasons for the research, advantages and disadvantages of taking part, and how data would be handled as part of the research. The same person then returned at least 24 h after this original approach to ask if the patient would like to be involved, to offer the opportunity to ask questions and to gain written consent. Twenty-nine people were approached to participate, with seven declining to be involved at this stage.

### Data Collection

Data collection took place between April 2022 and August 2023. Two technical instructors carried out the research interviews after receiving training from a member of the research team (AA). The structured schedule helped to maintain consistency between interviewers. Both interviewers were known to patients from their therapy and rehabilitation but did not have direct responsibility for student education. This prior relationship with patients was felt to be important to service user and carer members of the steering group who felt this would help with trust and honesty at a time of vulnerability for patients.

All interviews were carried out in person with one interviewer and one participant in private areas of inpatient wards. Participant responses were recorded in writing during the interview, using exact words and phrases where appropriate. Following discussion within research steering group meetings, it was decided not to audio-record interviews to minimise perceived burden or intrusion for participants. Interviews lasted 30 min on average. The period of data collection was stopped for pragmatic reasons when the placement periods during the 2023 academic year had come to an end.

### Data Analysis

Reflexive thematic analysis (14) was used to analyse data. Two analysts (GB and AK – representing both academic and carer members of the research team) initially independently coded the same sample of hand-written interview records and met to agree initial codes. Examples of initial codes included descriptive terms such as ‘consent’, ‘involvement’ and ‘explanation’. With consensus about initial codes, the remaining interview records were analysed independently by one analyst, but further meetings were used to add or amend codes and collapse codes into overarching themes.

All members of the research steering group were then involved in subsequent stages of analysis to reflect on the coherence of themes, the examples of data in support of themes and the alignment between themes and the research aim. During research meetings, the research team also revisited the decision to stop data collection, reflecting that we had reached an adequate level of data to develop understanding in relation to our aim. Due to time and resource constraints, there was no opportunity to go back to research participants to share themes.

Four themes were identified: *consent to work with multiple students; responses to working with multiple students; multiple students and feelings of safety; making connections with multiple students*.

## Findings

Twenty-two participants (including those involved at the pilot stage) gave consent and were interviewed for the study; 13 participants from Elderly Care services, five within Acute Medicine services and four within Neurorehabilitation. The majority of participants had worked with a group of physiotherapy students as part of their care although four had worked with a group of students consisting of occupational therapy and physiotherapy students.

Most patients were reflecting on the experience of working with two students at the same time (16), with four patients having experience of working with a group of three students together, one patient discussing the experience of working with a group of four and one patient was unable to recall.

### Consent to Work with Multiple Students

Patient experiences of being given advance notice about working with students suggested that this element of information sharing was not explicit. One patient discussed not being told directly in advance but assumed that students would be part of their care based on past experiences. Other patients discussed that they found out about student involvement and working with a group of students when they introduced themselves (P4, P7, P10, P21) or ‘when they turned up’ (P6).

Most patients reflected that at the start of an interaction, students did ask for consent and they understood the reasons for working with students, with reflections such as ‘they have to learn the job’ (P3) being a common theme. However, patients did not share any responses which suggested they understood the more detailed reasons why groups of students may be working collaboratively and involved in their care.

There were contrasting perspectives shared about whether people would have felt able to say no to working with a group of students. Some patients suggested they would have said no if they were not comfortable and one person gave an example of saying no on one occasion when they ‘were in a lot of pain’ (P4). However, a smaller number of patients did indicate that they did not feel they could say no to working with the group of students.

Reflecting on the fact that students are temporary and transient members of the healthcare team, patients were asked whether it was important to know how long they would be working with students and the majority indicated that this was not important to them. Those who did indicate that this was important associated this more with knowing if their therapy was going to continue, rather than knowing when students were approaching the end of a placement period.

### Responses to Working with Multiple Students

The majority of patients reported positive responses to both the idea of working with students before meeting them, and the experience of working with students as part of their care. Words such as ‘happy’ (P6, P7, P11, P16) and ‘good’ (P2, P4, P10, P15) were frequently used when describing their feelings about meeting a group of students. One patient shared a reflection about reciprocity, indicating they had a positive experience of working with the group of students and they ‘felt helpful to them’ (P21).

One patient acknowledged that they were worried initially about meeting students but feelings of worry eased when they met them because ‘they were nice people and [I] enjoyed having someone to talk to’ (P1). Another patient discussed how working with a group of students did not phase them as they had worked with students before.

### Multiple Students and Feelings of Safety

Most patients suggested feeling safe when working with students but importantly in the context of this study, the extracts suggest that people experienced feelings of safety which they attributed to working with a group. For example ‘there was a lot of them there if I had a wobble’ (2) and ‘having more hands-on deck helped me feel safer’ (P5).

Some patients were able to compare the experience of working with a group of students to a previous experience of working with one student, with responses indicating no perceived difference summarised by the extract ‘I felt safe to work with one student and it felt the same to work with two’ (P8).

When asked for their view on what the maximum number of students in a group should be, the majority of patients indicated they felt two would be the optimum number, with reasons such as it helps people to remember faces (P12), and more would feel impersonal (P17). However, responses to this question presented as being linked to the number of students people had actually worked with – for example, the patient who had worked with four students felt this would be an optimum number. One patient reflected that a maximum number would make little difference as they can ‘split up and watch each other’ (P10), suggesting their experience and feelings of safety are more related to how the learning is planned rather than being related to a specific group number.

### Making a Connection with Multiple Students

Overwhelmingly, comments about the quality of the relationships with students were positive, one patient reflecting that they enjoyed their company (P1) and another suggesting they ‘had a good laugh’ (P7). One patient suggested the students ‘were a lovely team’ (P5) which indicated a particular quality generated from working collaboratively as a group. Another patient suggested students went the extra mile for them (P10), and two other patients discussed how working with the students increased their confidence (P3, P4).

To offer contrast, one patient reflected that ‘sometimes I could not be bothered working with them’ (P13). This patient went on to suggest that their experience of working with students was ‘good…but sometimes I like to be left alone’. Another patient reflected that it was sometimes hard for them to follow conversations with lots of people in the room (P4).

## Discussion

This exploratory study presents important insights into the patient experience of working with multiple students, alongside patient perspectives of working with AHP students more generally – both under-explored areas in current research.

Informed consent is an integral part of conduct and ethical practice for all AHP students (15) and participant responses indicated that students did gain consent at the start of episodes of care. However, responses led to important reflections about giving consent to work with students. For patients in this study, consent may not always have been sought in advance and may have been sought by the students themselves on arrival, both potentially influencing whether people felt able to say no. While some AHP professional standards have specific statements about gaining consent to work with students (16), others do not make this explicit (17), and none have clear statements about who's responsibility it is to gain this consent to work with students and when it should be sought. This can be contrasted to the British Medical Association (BMA) guidance that the qualified practitioner responsible for medical students in the UK should gain consent for students to be present, and where possible this consent should be sought prior to the arrival of students (18). Of specific relevance to this study, it was not obvious to patients that they were consenting to intervention with multiple students, could choose one student or had the right to decline, either in advance or in real time.

An additional insight in relation to consent was that one participant linked giving consent to working with multiple students to their broader illness experience (P4 not giving consent when they were in a lot of pain). This example emphasises that the healthcare experience (in this case, working with a group of students) is part of an experience continuum, including experience of health, ill-health and disease (19). Understanding this was beyond the scope of this exploratory study although is an interesting area for further enquiry.

The positive responses to working with multiple students relating to feelings of safety are an important finding from this study. The science of human factors could suggest episodes of care involving teams of greater numbers, but with less experience and familiarity, could subsequently increase the likelihood of adverse events (20). And while this was not measured in a formal way in this current study, the reflection that patients felt safe, and in some instances ‘safer’, is significant to note.

Reflecting further on the insights that having more students involved felt safer or would be helpful in the event of a ‘wobble’, patients were perhaps perceiving greater numbers of people as a mitigating factor in the prevention of harm which is reported elsewhere as an attribute of patient safety (21). The notion of *presence* is also reported as an attribute of patient safety (22), particularly for hospitalised patients, and responses reflected the value of having multiple people available and close-by.

Existing literature suggests patients are generally positive about the presence of students (23) and reassuringly encounters with multiple students did not present as having a negative effect on patient experience. Indeed, an environment encouraging peer-learning, which empowers students to problem-solve and build confidence together (5), may have enhanced the patient experience of care, perhaps reflected in extracts such as students being ‘a lovely team’.

The extract from one patient that conversations could be difficult to follow is important for all health professionals and has potential links to patient safety. Over-crowded rooms with groups of students are noted elsewhere as being uncomfortable or challenging for patients (9). It is therefore essential to ensure communication is clear, information is not missed or misunderstood and importantly, the patient's voice remains central. Identifying who will take a leading role to communicate with the patient in each interaction could be a helpful strategy. Furthermore, the response from one patient that the actual number of students made little difference if they split up or observe helps to further emphasise the importance of intentional planning of roles.

Perceptions of optimum numbers of students presented as being closely aligned to the actual number of students patients had experienced working with – for example, if a patient had worked with two, the response was likely to identify two as an optimum number. Examples from published literature of peer-learning and collaborative placements also describe models with two students as the most common in AHP (5). Reflecting on this, it is important for AHPs not to remain fixed to familiar placement designs, to transfer promising evidence from other disciplines about collaborative models involving larger groups of students and to push the boundaries with innovative placement design. Patient experience should also become a central cornerstone of this design and evaluation.

## Limitations

This was a small-scale exploratory study involving three clinical specialisms in one acute NHS Trust. The approach of the lead educator and the culture of one organisation are likely to contribute to patient experience which could also have influenced findings. Larger evaluation involving wider contexts of practice and other healthcare students would all help to further understanding, particularly regarding transferability to wider settings. The majority of patients were reflecting on experiences of working with physiotherapy students, with smaller numbers involving occupational therapy and physiotherapy students working together. Additionally, the structured nature of the interview meant there were limited opportunities to develop deeper descriptions of experience.

## Conclusion

The findings of this small-scale qualitative study suggest that patients experienced positive relationships with multiple students and felt safe during care.

As numbers of students in practice areas increase in response to workforce and workplace demands, educators and students may question the impact on patients’ privacy, dignity, communication, safety and their overall experience. These findings should help to reassure educators and students that quality does not need to be compromised when more than one student is present.

Findings can also contribute to planning high-quality practice-based learning for multiple students. Addressing how and when consent is obtained from patients and being explicit where this involves groups of students could positively influence patient participation and student involvement in the delivery of quality care. HEIs can also help to prepare students for situations where they may be working in groups with multiple students – in both planned and unplanned ways – reinforcing the need to develop clear communication and identification of roles which is intentional and not left to chance.

## Supplemental Material

sj-docx-1-jpx-10.1177_23743735241241461 - Supplemental material for The Patient's Experience of Working with Multiple Allied Health Professional Students – A Qualitative Interview Study Supplemental material, sj-docx-1-jpx-10.1177_23743735241241461 for The Patient's Experience of Working with Multiple Allied Health Professional Students – A Qualitative Interview Study by Adele Anderson, Julie Morrow, Anita Knighton, Andrew Lloyd, Jane Noble and Gemma Bradley in Journal of Patient Experience
