# Supplementary material for: The Patient's Experience of Working with Multiple Allied Health Professional Students – A Qualitative Interview Study
Source: J Patient Exp. 2024 Apr 28;11:23743735241241461. doi: 10.1177/23743735241241461 (PMC11057343; doi:10.1177/23743735241241461)
Supplement: sj-docx-1-jpx-10.1177_23743735241241461 - Supplemental material for The Patient's Experience of Working with Multiple Allied Health Professional Students – A Qualitative Interview Study [file sj-docx-1-jpx-10.1177_23743735241241461.docx]

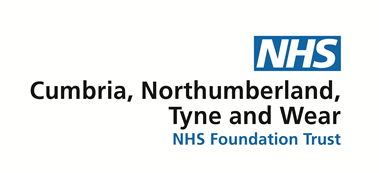


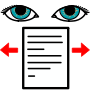


**Evaluating service user and carer perspectives of working with groups of students on placement**

You have recently received care from more than one occupational therapy or physiotherapy student.

We would like to ask you about this. We want to find out how people feel about the care they get from more than one student.

**We are not asking about each student’s skills and abilities.**

1.
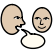
Did anyone tell you students might care for you?

How did you find out?

1.
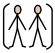
Did anyone tell you more than one student might care

for you at the same time?

How did you find out?


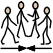

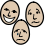


1. How did you feel when you met the students?


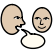

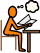


1. Did they tell you they were students?


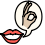


1. Did you agree to have students care for you?

Who asked you?


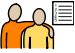


1. Did the students tell you what they would be doing with you?
2.
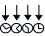
Did they tell you this every time they cared for you?

What did they say?

1.
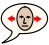
Did you feel you could say no to having students care for

you or ask for someone else?

1.
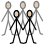
What was the highest number of students caring for you

at one time?


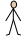


1. Have you ever worked with just one student in the past?


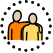
 Did it feel safe?

1.
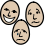

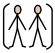
If you have worked with just one student before, did it

feel different having a group of students caring for you?

1.
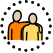
If you have never worked with just one student before,

did you feel safe with a group of students caring for you?

1. Did the group of students:


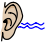
Listen to you?


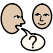
Ask you about your care?


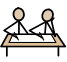
Involve you in decisions about your care?

Can you tell us more about how they did this?

1.
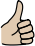

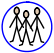
How well did you get on with them?
2.
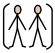

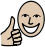
Did you enjoy working with two or more students?

Can you tell us why?

1.
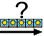
Is it important to know how long the students will care for you?

Why?

1. Was having a group of students caring for you good, bad or did it


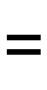

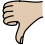

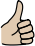
 not make a difference?

Why do you say that?

1.
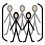

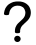
What do you think is the highest number of students

that should care for you at any one time?

Why?

1.
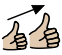
How could the care from a group of students be improved?
2.
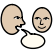

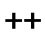
Is there anything else that you would like to tell us

about the care you got from the group of students?

**
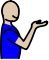
**

**Thank you for your time**
